# Multimodal combination of neuroimaging methods for localizing the epileptogenic zone in MR-negative epilepsy

**DOI:** 10.1038/s41598-022-19121-8

**Published:** 2022-09-07

**Authors:** Pavel Říha, Irena Doležalová, Radek Mareček, Martin Lamoš, Michaela Bartoňová, Martin Kojan, Michal Mikl, Martin Gajdoš, Lubomír Vojtíšek, Marek Bartoň, Ondřej Strýček, Martin Pail, Milan Brázdil, Ivan Rektor

**Affiliations:** 1 First Department of Neurology, St. Anne’s University Hospital and Faculty of Medicine, Masaryk University, Brno, Czech Republic; 2 Multimodal and Functional Neuroimaging Research Group, CEITEC-Central European Institute of Technology, Masaryk University, Brno, Czech Republic

**Keywords:** Epilepsy, Epilepsy, Biomedical engineering

## Abstract

The objective was to determine the optimal combination of multimodal imaging methods (IMs) for localizing the epileptogenic zone (EZ) in patients with MR-negative drug-resistant epilepsy. Data from 25 patients with MR-negative focal epilepsy (age 30 ± 10 years, 16M/9F) who underwent surgical resection of the EZ and from 110 healthy controls (age 31 ± 9 years; 56M/54F) were used to evaluate IMs based on 3T MRI, FDG-PET, HD-EEG, and SPECT. Patients with successful outcomes and/or positive histological findings were evaluated. From 38 IMs calculated per patient, 13 methods were selected by evaluating the mutual similarity of the methods and the accuracy of the EZ localization. The best results in postsurgical patients for EZ localization were found for ictal/ interictal SPECT (SISCOM), FDG-PET, arterial spin labeling (ASL), functional regional homogeneity (ReHo), gray matter volume (GMV), cortical thickness, HD electrical source imaging (ESI-HD), amplitude of low-frequency fluctuation (ALFF), diffusion tensor imaging, and kurtosis imaging. Combining IMs provides the method with the most accurate EZ identification in MR-negative epilepsy. The PET, SISCOM, and selected MRI-post-processing techniques are useful for EZ localization for surgical tailoring.

## Introduction

Drug-resistant epilepsy, found in approximately 30% of epileptic patients, represents a considerable health problem. Resection of the entire epileptogenic zone (EZ) is associated with a high chance of sustaining long-term seizure freedom^1,2^.

In the past, primary attention was paid to patients with an evident lesion on MRI, known as lesional cases. Patients without apparent MRI lesion, known as MR-negative or non-lesional cases, were not considered optimal surgical candidates. Thus, they were often left outside the main clinical interest and even not included in surgery programs in some epilepsy centers. However, this opinion has changed in the past decade, as more attention has been paid to MR-negative cases with satisfying results^3,4^.

The success of epilepsy surgery depends on the precise location and delineation of EZ^5^. The location is based on the careful analysis of aura, seizure semiology, distribution of interictal epileptiform discharges (IEDs), ictal video-EEG and high-quality 3T MRI; other methods and techniques can be also involved. Many advanced methods have been studied in recent years. These methods are focused on both structural and functional information. The satisfactory accuracy of several methods and their clinical utility have been demonstrated^6–10^.

The epileptogenic cerebral cortex differs from healthy brain tissue on microscopic and macroscopic levels. Differences in the cerebral cortex properties can be anticipated when analyzing advanced methods such as MRI, EEG, PET, and SPECT. Structural differences can be revealed by analyzing gray matter (*gray matter volume*, *gray matter concentration*), thickness of the cortex (*cortical thickness*), blurring of the transition between gray and white matter (*junction*), and the displacement of gray matter (*normalized FLAIR*, *gray matter volume*, *white matter volume*, *surface-based methods*). Changes in the brain microstructure are related to different distributions of water molecules and different diffusion coefficients (*mean diffusivity*, *axial diffusivity*, *radial diffusivity*, *fractional anisotropy*, *mean kurtosis*, *axial kurtosis*,* radial kurtosis*). Pathological tissue influences physiological processes in the brain at the local and global levels. Local changes can be represented by decreased metabolism (*PET*) and reduced blood perfusion (*arterial spin labeling*, *SPECT*) or by a change of electrical activity in the EZ (*electrical source imaging*, *local synchrony*). Global changes are reflected by network disruptions and their fluctuations (*amplitude of low-frequency fluctuation*, *regional homogeneity*).

Each of these methods can provide a piece of the knowledge puzzle in EZ location and delineation, but we still lack a direct comparison among individual methods in the same patient groups in terms of their accuracy. Due to the huge amount of concordant and discordant results of these techniques, it is difficult to choose the optimal combination of methods for clinical practice. Patients can be bothered by lengthy or repeated scanning, and the financial costs associated with prolonged use of MRI scanners cannot be overlooked.

These considerations inspired us to conduct the present study, in which we address the problem of identifying the optimal combination of neuroimaging techniques in evaluating patients with MR-negative drug-resistant focal epilepsy. We employed a wide range of different methods to consider the localization of EZ from different perspectives. We did not expect the same or completely overlapping results for all methods, because the methods are based on different EZ characteristics. However, we expected that most of this overlap would be present in the EZ itself.

## Methods

The project is focused on available epilepsy imaging methods (IMs) that may serve for identifying the EZ and on their benefits in the diagnostic process in patients with MR-negative drug-resistant epilepsy. Based on literature published in the last 20 years^6–11^ and on widely used methods, we identified the IMs potentially best suited for epilepsy diagnosis. The measurement protocol was designed in 2016 using the PET, SPECT, advanced MRI, and EEG methods reported as useful for lesion identification, which we refer to here as the general battery. Detailed sequence settings are in the supplementary materials.

Patients were prospectively recruited in the tertiary Brno Epilepsy Center. All patients referred as epilepsy surgery candidates with MR-negative drug-resistant epilepsy to the Epilepsy Department in St. Anne’s University Hospital in Brno between 2016 and 2019 were indicated for the study protocol. Patients who underwent successful resection surgery of the EZ were included in this study.

We focused on the comparison of IMs and their optimal combination for surgical planning. Our study targeted three objectives:

- **Objective 1:** To establish **a fundamental battery** of IMs for evaluating MR-negative patients. We reduced the general battery by evaluating the mutual similarity of the methods and the accuracy of the EZ localization.
- **Objective 2:** To compare individual IMs in the fundamental battery in terms of ***accuracy*** as confirmed by EZ resection.
- **Objective 3:** To define the optimal combination of IMs for localizing EZ in MR-negative patients.

The study was approved by ethics committee of the Masaryk University. All patients gave their written informed consent before the study started. All experiments were performed in accordance with relevant guidelines and regulations and meet the Declaration of Helsinki. All methods were carried out in accordance with relevant guidelines and regulations.

### Epilepsy imaging methods (IMs)

The **selection criteria** for advanced neuroimaging were as follows: (1) methods based on MRI, 18FDG-PET, ictal/interictal SPECT, or high-density EEG postprocessing; (2) covering the whole brain; and (3) enabling voxel property calculation. Surface methods (marked with an asterisk (*)) were completely processed in the surface space until the results were resampled^12^ into volumes to allow comparisons with the surgery mask.

We identified 38 IMs based on MRI, 18FDG-PET, SPECT, and EEG postprocessing; these formed the **general battery** and are summarized in Table 1. An example of all methods in one patient is shown in supplementary materials S5.

**Table 1:** Summary of 38 imaging methods identified in the literature.

	Type	Name	Acronym	Description
MRI-IM	Structural (T1W, FLAIR)	Gray matter volume	GMV	The GMV/GMC reveals changes in gray matter (GM) volume or signal intensity, i.e., atrophy, GM displacement or abnormal geometry of gyrification^13^
		Gray matter concentration	GMC	
		Junction	Junction	Junction can identify subtle blurring between GM and WM, which is present in approximately 80% of cases with initially negative MR and subsequently proved focal cortical dysplasia (FCD)^14^
		Cortical thickness*	Thick*	Surface-based metrics measuring abnormalities in cortical thickness, sulcal depth, absolute mean curvature, or a fractal dimension of cortex^15,16^
		Sulcal depth*	SclD*	
		Gyrification index*	Gyr*	
		Cortical complexity*	Crtx*	
		White matter volume	WMV	WMV/WMC measures changes in WM volume or signal intensity^13^
		White matter concentration	WMC	
		Normalized FLAIR	FLR	Voxel-based analysis of normalized intensity in FLAIR scans compared to healthy control (HC)^17^
	PCASL	Arterial spin labeling	ASLhc	ASL measures quantitative cerebral blood flow (qCBF) using MRI. Results are compared to HC (ASLhc) or between right and left hemisphere(ASLai)^18–20^
		ASL asymmetry index	ASLai	
	FMRI-rest	Amplitude of low-frequency fluctuation	ALFF1 ALFF2 ALFF3 ALFF4 ALFF5	ALFF/fALFF detects low-frequency fluctuations during spontaneous activity which can be associated with the interictal epileptic discharges. fALFF uses global signal normalization^21^. Numbers 1 to 5 indicate the frequency band; details are in the supplementary materials
		Fractional ALFF	fALFF1 fALFF2 fALFF3 fALFF4 fALFF5	
		Regional homogeneity	ReHo	ReHo is a measure of similarity of BOLD signal fluctuation in a defined region^22^
	Diffusion	Mean diffusivity	MD	The diffusion is sensitive to the extent and direction of water molecule diffusion and provides unique information about tissue microstructure. MD measures the average water diffusion properties within a voxel. AD measures water diffusion in the direction of the highest diffusion. RD measures water diffusion averaged in the plane perpendicular to the direction of the highest diffusion. FA measures the degree of anisotropy in the diffusion tensor (0 means that diffusion is unrestricted or restricted equally in all directions, 1 means that diffusion occurs only along one axis and is fully restricted along all other directions^23^
		Axial diffusivity	AD	
		Radial diffusivity	RD	
		Fractional anisotropy	FA	
		Mean kurtosis	MK	Changes in kurtosis (statistical metric for quantifying the degree of difference between diffusion in pure fluids and diffusion in biological tissue) compared to HC^24^
		Axial kurtosis	AK	
		Radial kurtosis	RK	
PET-IM	NA	Fluorodeoxyglucose (FDG) PET	PEThc	Reveals a hypometabolism in the epileptogenic zone (EZ) compared to HC (PEThc) or contralateral hemisphere (PETai)^20,25^
		PET asymmetry index	PETai	
SPECT-IM	NA	SISCOM	SISCOM	SISCOM shows the difference between ictal and interictal SPECT = perfusion change during epileptic activity. STATISCOM and ISAS measure ictal perfusion compared to HC^26^
		STATISCOM	STATISCOM	
		ISAS	ISAS	
EEG-IM	IEDsD	HD electrical source imaging	ESI-HD	ESI is a well-established approach for EZ localization according to expertly labelled IEDs (IEDsD = IEDs dependent) in scalp EEG. The ESI basis is the projection of scalp EEG into brain volume^27^. ESI-HD is calculated using high density (HD) 256 electrodes
		Electrical source imaging	ESI-10–20	ESI calculated with electrodes placed according to a routine 10–20 system
	IEDsI	Local synchronization	LocSyn	LocSyn reveals regions showing increased local synchrony in neural activity (relative to HC) measured in scalp EEG^28^ (IEDsI = IEDs independent)

All IMs were measured and processed according to the state of art, including quality control. Methods with insufficient quality were not included in the study. The individual methods in detail, including sequence settings and detailed technical postprocessing, are described in the Supplementary Materials.

### Patient recruitment

***MR-negative epilepsy*** was defined as epilepsy with a negative or inconclusive finding of lesions using the 3T MRI presurgical epilepsy protocol, evaluated by a specialized neuroradiologist and discussed with the epilepsy surgery commission of the Brno Epilepsy Center. Standard clinical MRI sequences used for categorization are listed in the supplementary materials S3.

In all patients, the presurgical evaluation protocol preceded their inclusion in the project. The evaluation involved specialized epilepsy 3T MRI, long-term interictal and ictal video EEG, neuropsychological examination, 18FDG-PET, and ictal/interictal SPECT.

We enrolled 25 operated MR-negative drug-resistant patients. The patient group had two subgroups of patients: the first subgroup (12 patients) was defined by successful surgery with ILAE 1 or 2, and the second subgroup (13 patients) was included on the basis of epileptogenic histopathological findings (FCD or hippocampal sclerosis). In total, our IM protocol was applied to 116 patients, of which 27 underwent resection surgery; in 2 of those 27 patients, the outcome was without decreased seizures and with inconclusive histological findings, and therefore they were not included in this study. Demographic data are summarized in Table 2 and individual patient characteristics are shown in supplementary materials S1.

**Table 2:** Demographic data of investigated patients.

Parameter	Value
**Patient group size**	25 patients
**Age at the surgery/measurement** Median (years)	30 (min–max, 17–50)
**Sex**	16 males, 9 females
**Localization of surgery** Side Lobe	18 right, 7 left TLE in 9 patients, FLE in 10, ILE in 2, OE in 4
**Outcome according to ILAE classification**	11 × ILAE 1, 1 × ILAE 2, 2 × ILAE 3, 9 × ILAE 4, 2 × ILAE 5 Inclusion of ILAE class 3–5 was based on epileptogenic histopathology finding
**Histology**	15 × FCD (2 × IA, 1 × IB, 8 × IIA, 2 × IIB, 1 × IIIA, 1 × unclear), 5 × HS, 4 × negative, 1 × meningioangiomatosis Patients with negative histological findings were included because of positive surgical outcomes
**Surgery size** median (cm^3^)	21 (min–max, 4–52)
**Age at epilepsy onset** Median (years)	9 (min–max, 0–26)
**Duration of epilepsy before surgery/measurement** Median (years)	21 (min–max, 3–47)

*FCD* focal cortical dysplasia, *FLE* frontal lobe epilepsy, *HS* hippocampal sclerosis, *ILE* insular epilepsy, *ILAE* International League Against Epilepsy, *max* maximum, *min* minimum, *PE* parietal epilepsy, *OE* occipital epilepsy, *PLE* parietal lobe epilepsy, *TLE* temporal lobe epilepsy.

For each patient, we created resection masks for the IM localization accuracy evaluation process. These resection masks were based on resection borders in post-resection imaging.

### Incomplete data

**Figure 1:**
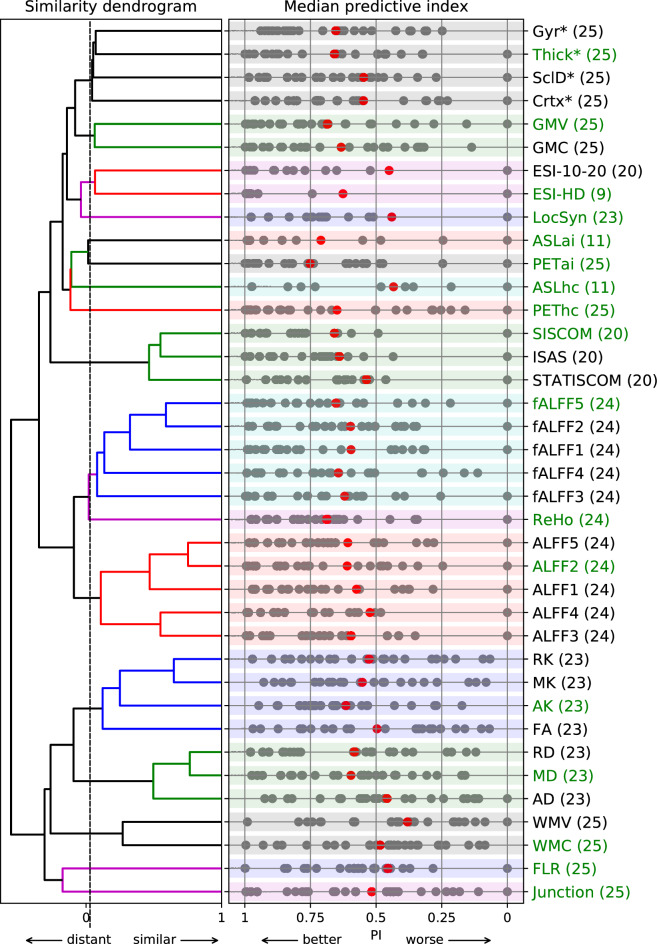
The selection of methods for the fundamental battery. Hierarchical dendrogram is plotted on the left side; threshold for imaging method selection is indicated by a vertical dashed line. Red dots show the mean predictive index. The numbers in parentheses after the method indicate the number of completed measurements of that method; abbreviations of methods are presented in Table 1. The battery (n = 38) was reduced to 17 methods that formed the fundamental battery and that are marked in green.

For technical reasons, some methods were not available for all patients (numbers are shown in Fig. 1). The biggest challenges were associated with ESI, SPECT, and ASL.

- We did not evaluate ESI-HD in 16 out of 25 patients and we did not evaluate ESI-10-20 in 5 out of 25 patients because of IED absence during recording;
- the SPECT was not completed in 5 out of 25 patients because of problems obtaining ictal scans;
- ASL was completed in 11 out of 25 patients because we updated to an improved protocol during the study (see supplementary materials S2.2).
- In other IMs, we have at least 90% of the measured data for each method and an average 96% measured data in whole battery.

### Healthy controls

The principle of pathological tissue detection in most IMs is based on a statistical comparison with a healthy population. This applies to MR-Structural, MR-Fmri, MR-Diffusion, MR-ASLhc, PEThc, STATISCOM, ISAS, and LocSyn. Conversely, healthy controls are not used for ASLai, PETai, SISCOM, or ESI.

We used 110 age-matched (median age 31, min–max 18–59 years; 56M/54F) healthy controls (HC) for all of the above MR and EEG IMs; HC were measured by the same protocol as the patients.

Because we cannot measure nuclear imaging in healthy patients, separate control groups were used for PEThc, STATISCOM, and ISAS. For PEThc, we used 24 control subjects recruited from oncological patients whose findings on their head FDG-PET were assessed as normal. They had normal MR scans and no neurological nor psychiatric diagnoses. For STATISCOM and ISAS, 14 ictal-interictal SPECT image pairs from healthy controls were downloaded from the Healthy Normal Database on http://spect.yale.edu/downloads.html.

### The comparison of individual IMs

#### Methods accuracy

We thresholded each IM result with the 90th percentile into a binary map. This means that we selected the strongest result corresponding to 10% of the whole brain volume from each method for further analyses. The choice of the percentile is arbitrary; we considered the 80th, 90th, and 95th percentiles; the 90th achieves the highest mean accuracy across the entire group.

***Sensitivity*** was calculated based on thresholded images of IM results and resection masks. The surviving voxels were separated into two groups and counted: the number of voxels inside the resection mask—*true positive (TP)* and the number of voxels outside the resection mask—*false positive*. The whole resection mask is *positive (P)*.

$$Sensitivity = SEN= \frac{TP}{P}$$

Based on sensitivity, we calculated the ***Predictive Index**** (PI)* using a significance exact test with K = 1000 random surgery masks. They were generated to have a random location within the brain volume and a size randomly chosen from the distribution of mask sizes over the surgery group. Then, *PI* is defined as

$$PI= 1 -\mathrm{ p}\left(SEN > SE{N}_{0}\right)=1 - \frac{1}{K}{\sum }_{k=1}^{K}I\left(SE{N}_{k} > SE{N}_{0}\right)$$

where *I* is the indicator function, *SEN*_*0*_ is the observed sensitivity value with the actual surgery mask, and *SEN*_*k*_ are sensitivities calculated from random masks.

The *PI* expresses an accuracy of EZ localization and takes values from 0 to 1, with 1 corresponding to the best match with EZ. Thanks to the exact test, PI effectively includes both sensitivity and specificity.

#### Methods similarity

For each uniformly 90th-percentile binary thresholded pair of IMs *a*_*90*_ and *b*_*90*_, the mutual similarity (MS) was determined to be

$$MS\left(a,b\right)=\frac{\left|{a}_{90}\cap {b}_{90}\right|}{{N}_{90}}$$

where N_90_ is the number of 90th-percentile voxels above threshold (N_90_ =|a_90_| =|b_90_|). Similarities between the same methods through the patient group were averaged.

### Objective 1—a fundamental battery for advanced neuroimaging

In this objective, we intended to reduce the number of methods and to formulate a **fundamental battery** selected based on two criteria – similarity and accuracy.

- **Similarity**—We selected the methods with a low grade of overlapping results.
- **Accuracy**—If two or more IMs were similar, we gave priority to the method with the highest PI.

We used hierarchical clustering based on mutual similarity to divide methods into several clusters so that each cluster comprised methods that provide similar results. The fundamental battery was designed to reasonably represent all clusters and to cover all modalities generally used in presurgical evaluation.

### Objective 2—comparison of IMs in a fundamental battery

The main aim was to compare the accuracy of individual IMs from the fundamental battery in the whole surgical group.

First of all, the difference in the accuracies of individual IMs was evaluated using the Kruskal–Wallis test to verify that it made sense to deal with rankings. The mean PI for each method was then calculated and IM methods were sorted according to their mean PI. In addition, we performed a Wilcoxon signed-rank test for each pair of IMs, with FDR for multiple comparison correction, to show which methods differed significantly in accuracy.

### Objective 3—the optimal composition of IMs for localizing EZ

In the previous objective, a sorted list of non-duplicated IMs was created. The initial methods are more accurate, and the subsequent methods are less accurate. For clinical use, we recommend selecting a suitable number of initial methods, but the choice of a reasonable number of methods is a trade-off between accuracy and efficiency. The number of recommended methods can be deduced using Reverse Helmert coding, which finds positions from which the subsequent methods are statistically less accurate than the previous ones.

Implementation of a Helmert contrast for linear models in R was used. PI was a dependent variable and individual IMs were explanatory variables.

### Ethical approval

The study was approved by ethics committees of the participating institutions and all patients gave their informed consent before the study started. All methods were carried out in accordance with relevant guidelines and regulations.

## Results

### Objective 1—proposition of a fundamental battery for advanced neuroimaging

Seventeen diverse clusters (about half of all methods) representing the entire general battery (N = 38) was identified according to the similarity clustering. A whole hierarchical dendrogram is plotted on the left side of Fig. 1 and the threshold for IM selection is indicated by a vertical dashed line.

In the next step, one IM with the highest median PI was selected from each cluster. Finally, Thick*, GMV, LocSyn, ESI-HD, ASLai, PETai, ASLhc, PEThc, SISCOM, fALFF5, ReHo, ALFF2, AK, MD, WMC, FLR, and Junction were selected.

The methods that were not selected from the general battery were usually slightly different versions of selected methods from the same source data with lower PI.

### Objective 2—comparison of individual IMs in fundamental battery

The differences between the individual IMs in the fundamental battery are statistically significant with a p value = 0.006.

**Figure 2:**
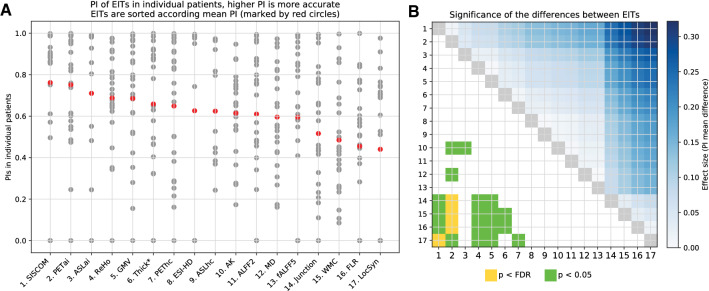
Mean Predictive Index of individual neuroimaging techniques (IMs). (**A**) The predictive index (PI) was calculated for each IM in each patient. Abbreviations of methods are presented in Table 1. For each IM, PIs were averaged, and the IMs were sorted according to mean PI (red points; a higher PI indicates better results for a given IM). (**B**) Matrix showing effect size of comparison of each pair in IMs. At the top is the difference in mean PI (a darker blue indicates a larger difference); at the bottom is the statistical significance for each comparison (uncorrected p < 0.05 and after FDR correction). The numeric labels indicating methods correspond with the order in Part A.

When all IMs were sorted based on their mean Predictive Index, we obtained the following order: (1) SISCOM, (2) PETai, (3) ASLai, (4) ReHo, (5) GMV, (6) Thick*, (7) PEThc, (8) ESI-HD, (9) ASLhc, (10) AK, (11) ALFF2, (12) MD, (13) fALFF5, (14) Junction, (15) WMC, (16) FLR, and (17) LocSyn; details are in the left part of Fig. 2.

The right part of Fig. 2 indicates which pairs of IMs differ significantly from each other. Green square indicates uncorrected p-value lower than 0.05 and yellow squares indicates FDR-corrected p value lower than 0.0014.

### Objective 3—optimal composition of IMs for localizing EZ

The statistical difference of the neighboring methods in the sorted list was compared using the Helmert contrast. The first 13 consecutive methods were not significantly different; the change in accuracy occurs at the 14th junction, which is significantly less accurate than the previous ones (with p = 0.02).

Based on these results, it seems reasonable to perform the first 13 leading methods from this ranking in all patients with MR-negative epilepsy: (1) SISCOM, (2) PETai, (3) ASLai, (4) ReHo, (5) GMV, (6) Thick*, (7) PEThc, (8) ESI-HD, (9) ASLhc, (10) AK, (11) ALFF2, (12) MD, and (13) fALFF5.

The required MRI sequences are as follows: T1W, Diffusion, fMRI, and ASL. Total acquisition time for all MRI sequences is about 40 min.

## Discussion

The success of epilepsy surgery is based on the precise localization of the EZ, which can be relatively straightforward in patients with lesional MR^29^. By contrast, EZ localization and delineation can be extremely complicated in patients with MR-negative epilepsy, especially with extratemporal epilepsy. The patients with MR-negative epilepsy form approximately one third of the surgical series, in most cases due to focal cortical dysplasia (FCD) lesions that were not detected on MRI^16^. In our study group, 15 patients were revealed to have FCD after surgery. In 5 patients, hippocampal sclerosis undetected by MRI and in 4 patients negative histopathological findings with good surgical outcomes were revealed.

In terms of imaging development, the IMs used in this study are composed of traditional as well as recently introduced methods. Traditional methods include well-established methods such as FDG-PET and SISCOM^30–32^. The clinical utility of these methods has been proven. However, there are still patients for whom these traditional methods fail and even their acquisition can be complicated (for example, due to the necessity of radiotracer application or application at the beginning of a seizure). From this point of view, it seems reasonable to focus on the development of novel techniques based on MRI and HD-EEG postprocessing.

In our project, based on published data we identified 38 techniques that could be helpful in EZ delineation. Despite the high number of published techniques and expert comparisons of several methods^33^, we did not find any publication related to the quantitative comparison of this number of different neuroimaging techniques. To the best of our knowledge, the presented study is the first to quantitatively compare the wide spectrum of different neuroimaging techniques for EZ localization in patients with MR-negative epilepsy.

The target of this study is to define an optimal battery of IMs for clinical praxis in MR negative focal epilepsies. The battery selected according to this comparison is recommended for the presurgical localization of the EZ.

Traditional methods such as FDG-PET and SISCOM are in the first positions of the recommended battery. Based on the literature, PET seems to provide valuable information for EZ localization. The mean detection rate in 11 studies reporting on epilepsy surgery candidates was 71% (ranging from 36 to 93%)^34^. There were significant differences between temporal and extratemporal epilepsy. In temporal epilepsy, the value of PET is extremely high, with a sensitivity of 86% and false-localization rates of 3%^30^. In extratemporal epilepsy, hypometabolism can mimic temporal-like patterns, i.e. it can be more widespread and involve temporal areas^30^. SISCOM reflects the dynamics in blood perfusion associated with the transition from an interictal to an ictal state. A limitation of ictal SPECT is the necessity of administering a radiotracer immediately after seizure onset. Chen and Guo identified 11 studies with 320 patients; 270 patients were SISCOM positive. In 142 MR-negative patients, the SISCOM positive rate was 83.8%^31^.

ASL and ReHo had positive results, on the third and the fourth position. ASL is an MRI perfusion technique that enables the quantification of cerebral blood flow. Recent studies demonstrated a high efficacy of ASL^19^ and similar results of PET and ASL in localizing EZ in MR negative epilepsy^20^. The important advantage of ASL over FDG-PET is its independence from the application of a radioactive tracer, rapid data acquisition within the spectrum of clinical MR recorded in epilepsy patients, and lower costs^35^. In our study, ASL demonstrated good results; however, the number of patients with ASL was 11. The number was limited due to a change of the protocol during the research. The usefulness of ASL should be confirmed by larger cohorts. ReHo is a method derived from resting-state fMRI that explores the synchronization of activity in different brain regions^22^. The alternation of this synchronization was found in several neurological disorders including epilepsy^21^. The usefulness of ReHo for surgical tailoring was not systematically studied, but its possible value was suggested^36^.

These methods are followed by structural MRI techniques, namely voxel-based morphometry GMV, and surface-based Thick* techniques. Although the usefulness of voxel-based morphometry was verified in patients with MR-negative epilepsy^37^, these techniques also have some limitations, such as the absence of spatial relationships across the cortical surface, and a high susceptibility to any faults in acquisition^38–41^. To overcome these limitations, surface-based methods were proposed^15,42^. In our study, the most successful surface-based methods was Thick*, which analyzes the cortical thickness.

ESI-HD derived from surface HD EEG, even though extremely promising in previous studies^43,44^, is placed in the middle of the fundamental battery. This result was probably conditioned by two factors. First, we included all patients in the surgical group with variable frequencies of IEDs. Second, the EEG recording time was limited (40 min). To obtain satisfying ESI results, it is probably necessary to preselect indicated candidates based on their IED frequency and/or to extend the recording time. We recommend using it in patients with high frequencies of IEDs. We also analyzed routine EEG ESI-10–20 with scalp electrode placement based on a 10–20 system. The results of this methods were similar (i.e., similar positions in a dendrogram) as ESI-HD, but accuracy was lower; ESI-10–20 was therefore not selected for the recommended battery. The routine ESI-10–20 contains a higher number of IEDs for analysis, but the density of scalp cover is much lower than in HD EEG.

Diffusivity and kurtosis methods were placed at the end of our ranking. Although many authors have reported good results^45–47^, this was not confirmed in our voxel-wise comparison. A possible explanation is that microstructure alterations in DTI and DKI metrics in MR-negative epilepsy are usually more widespread and not restricted only to EZ areas. We assume that approaches that combine multiple diffusion metrics^48^ or use tractography analysis would be advantageous. However, those methods did not allow the whole brain voxel property calculation and therefore were not included in our comparison due to the inclusive criteria.

The clinical application of our work is a comparison of the similarity and accuracy of methods, which makes possible to choose the most suitable methods for diagnosing patients with MR negative focal epilepsy. The selected methods provide a suitable basis for automatic fusion using mathematical approaches^32^ and machine learning. However, a higher number of patients in the training group is a prerequisite for an effective machine learning approach.

The basic limitation of our work is the patient group consisting of 25 heterogeneity operated patients. We included both successfully operated patients with outcomes defined as ILAE 1 and 2 and patients with epileptogenic histopathological findings (FCD or hippocampal sclerosis) in whom the effect of surgery was limited by the extent and location of the epileptogenic zone. Further confirmation on larger sample sizes will be needed.

The technical limitation of the study is the disparity in the compared methods. We compared a wide range of methods that cover the brain differently (surface-based methods are naturally limited to the cortex) and with different resolutions (EEG and nuclear methods achieve lower resolutions than MR methods). However, as far as we know, a more suitable comparison method has not yet been published and we consider the presented results to be beneficial for clinical practice. Other limitations are the relatively short time of EEG recording, and the absence of some sequences in some individuals.

The advanced MR methods recommended by our study are accessible and could be used in most centers. The duration of MR recording is approximately 40 min in 3T MR, making the proposed combination of methods utilizable in most epilepsy patients.

### Conclusion

A high number of IMs, based on MRI, SPECT, and PET post-processing, can be employed in the neuroimaging of MR-negative epileptic patients. We compared these IMs in patients after resective surgery and recommend an optimal multimodal combination of IMs for clinical use. Based on our results, we recommend the combination of PET, SISCOM, and advanced MRI methods (specifically SISCOM, PETai, ASLai, ReHo, GMV, Thick*, PEThc, ESI-HD, ASLhc, AK, ALFF2, MD, and fALFF5) for clinical use in patients with MR-negative epilepsy.

## Supplementary Information

Supplementary Information.

## Data Availability

The datasets generated and analysed during the current study are available in the MAFIL laboratory repository in Central Institute of Technology Masaryk University, Brno; contact ivan.rektor@ceitec.muni.cz.
