# Supplementary material for: Multimodal combination of neuroimaging methods for localizing the epileptogenic zone in MR-negative epilepsy
Source: Sci Rep. 2022 Sep 7;12:15158. doi: 10.1038/s41598-022-19121-8 (PMC9452535; doi:10.1038/s41598-022-19121-8)
Supplement: Supplementary file 1 — Supplementary Information. [file 41598_2022_19121_MOESM1_ESM.docx]

Multimodal combination of neuroimaging methods for localizing the epileptogenic zone in MR-negative epilepsy

Říha Pavel^1,2^, Doležalová Irena^1^, Mareček Radek^2^, Lamoš Martin^2^, Bartoňová Michaela^1,2^, Kojan Martin^1,2^, Mikl Michal^2^, Gajdoš Martin^2^, Lubomír Vojtíšek^2^, Bartoň Marek^2^, Strýček Ondřej^1^, Pail Martin^1^, Brázdil Milan^1,2^, Rektor Ivan^1,2^

^1^ First Department of Neurology, St. Anne’s University Hospital and Faculty of Medicine, Masaryk University, Brno, Czech Republic

^2^CEITEC-Central European Institute of Technology, Multimodal and Functional Neuroimaging Research Group, Masaryk University, Brno, Czech Republic

**Corresponding author:** Ivan Rektor, Brno Epilepsy Center and Central European Institute of Technology (CEITEC), Masaryk University, Kamenice 753/5, 625 00 Brno, Czech Republic. Tel: +420603864046, Email: [ivan.rektor@fnusa.cz](mailto:ivan.rektor@fnusa.cz)

Supplementary materials

[S1 Individual characteristics of patients 2](#_Toc110348777)

[S2 IMs technical description 3](#_Toc110348778)

[2.1 MRI-IM: Structural (GMV, GMC, WMV, WMC, Junction, FLR, Thick*, Gyr*, SclD*, Crtx*) 3](#_Toc110348779)

[2.2 MRI-IM: PCASL (ASLai, ASLhc) 4](#_Toc110348780)

[2.3 MRI-IM: fMRI-rest (ALFFx, fALFFx, ReHoKCC) 4](#_Toc110348781)

[2.4 MRI-IM: Diffusions (MD, AD, RD, FA, MK, AK, RK, MDK) 5](#_Toc110348782)

[2.5 PET-IM (PEThc, PETai) 6](#_Toc110348783)

[2.6 SPECT-IM (SISCOM, ISAS, STATISCOM) 7](#_Toc110348784)

[2.7 EEG-IM: IEDs-dependent (ESI-HD) 8](#_Toc110348785)

[2.8 EEG-IM: IEDs-dependent (ESI-10-20) 8](#_Toc110348786)

[2.9 EEG-IM: IEDs-independent (LocSyn) 9](#_Toc110348787)

[S3 Clinical sequences parameters 9](#_Toc110348788)

[S4 Specificity 10](#_Toc110348789)

[S5 Imaging methods (IMs) examples 10](#_Toc110348790)

# Individual characteristics of patients

| Patient | Surgery target | Type | Surgery size (cm^3^) | ILAE | Histology | Onset (year) | Sex | Age |
| --- | --- | --- | --- | --- | --- | --- | --- | --- |
| 01 | R AMTR, F cortectomy | AMTR, cortectomy | 52 | 5 | FCD IIIA (HS + FCD) | 4 | M | 30 |
| 02 | R frontal (SMA) | cortectomy | 4 | 1 | FCD IIA | 26 | F | 29 |
| 03 | L middle inferior frontal | cortectomy | 6 | 3 | FCD | 7 | M | 19 |
| 04 | R AMTR | AMTR | 14 | 1 | HS | 6 | F | 26 |
| 05 | R Frontal | cortectomy | 21 | 1 | FCD IIA | 10 | F | 24 |
| 06 | R frontoorbital | cortectomy | 17 | 1 | negative | 10 | M | 26 |
| 07 | L frontal operculum | cortectomy | 8 | 3 | FCD IIA | 15 | M | 21 |
| 08 | R insula | cortectomy | 26 | 4 | FCD IA | 6 | M | 17 |
| 09 | R temporal lateral | cortectomy | 18 | 1 | FCD IIB | 12 | M | 28 |
| 10 | R frontal | cortectomy | 11 | 4 | FCD IIA | 16 | F | 34 |
| 11 | L occipital parasagital | cortectomy | 15 | 2 | negative | 14 | M | 26 |
| 12 | R frontal | cortectomy | 8 | 4 | FCD IIA | 3 | M | 19 |
| 13 | R AMTR | AMTR | 30 | 1 | negative | 20 | F | 49 |
| 14 | R insula | cortectomy | 18 | 4 | FCD IB | 6 | M | 46 |
| 15 | L AMTR | AMTR | 24 | 1 | HS I | 5 | F | 24 |
| 16 | R frontal | cortectomy | 23 | 1 | FCD IIB | 3 | M | 25 |
| 17 | R AMTR | AMTR | 32 | 4 | HS | 13 | F | 22 |
| 18 | R temporal lateral | cortectomy | 23 | 1 | FCD IIA | 0 | M | 25 |
| 19 | R parieto-occipital | cortectomy | 6 | 5 | FCD IIA | 1 | M | 45 |
| 20 | R AMTR | AMTR | 23 | 4 | HS I | 15 | M | 27 |
| 21 | L fronto parietal | cortectomy | 16 | 4 | FCD IA | 8 | F | 30 |
| 22 | R frontal | cortectomy | 41 | 4 | FCD IIA | 12 | M | 31 |
| 23 | L temporo occipital | AMTR | 29 | 4 | Meningio angiomatosis | 12 | F | 33 |
| 24 | R AMTR | AMTR | 29 | 1 | HS I | 3 | M | 50 |
| 25 | L AMTR | AMTR | 25 | 1 | negative | 4 | M | 43 |
|  | Not included in the study | | | | | | | |
| N1 | R insula | cortectomy | 14 | 4 | negative | 8 | F | 24 |
| N2 | L frontal | cortectomy | 21 | 3 | negative | 29 | M | 35 |

# IMs technical description

The following section describes in detail the technical processing of all IMs used in the article. Including used machines, setting of acquisition parameters, data preprocessing and final statistical analysis.

## MRI-IM: Structural (GMV, GMC, WMV, WMC, Junction, FLR, Thick*, Gyr*, SclD*, Crtx*)

**Data acquisition**

MRI acquisition was performed on a 3T scanner Siemens Prisma using a multichannel head coil. The MRI protocol for voxel-based morphometry included 3D T1-weighted magnetization prepared rapid gradient echo (MPRAGE) sequence with TR = 2.3 s, TE = 2.33 ms, TI = 0.9 s, FA = 8°, isometric voxel size 1 mm in FOV 256 × 260 mm, 240 slices and FLAIR sequence with TR 6000 ms, TE 387 ms, TI1 1900 ms and isometric voxel size 1 mm in FOV 256 x 256 mm, 192 slices.

**Data processing**

Anatomical MRI data were analysed using SPM12 (www.fil.ion.ucl.ac.uk) and CAT12 toolbox (www.neuro.uni-jena.de/cat) running in Matlab R2017b.

Individual data were adjusted for spatial inhomogeneity with an intensity normalization filter and then denoised with the Non-Local Means (SANLM) denoising filter. High resolution data were segmented into gray matter (GM) and white matter (WM) using the SPM Tissue Probability Map (TPM) and registered into common MNI space using shooting template IXI555_MNI152_GS.

The preprocessing quality was assessed by a visual inspection of T1w and FLAIR scans for motion artifacts, low SNR, and other artifacts. The normalized GM maps were manually checked to verify they were correctly transformed into the MNI space, and subsequently the homogeneity of GM maps was automatically checked using the CAT12 tool Check Sample Homogeneity.

**Parameter’s map**

- GMC and WMC were calculated from spatially normalized (unmodulated) GM and WM maps (using SPM12) smoothed with 6 mm FWHM isotropic Gaussian kernel.
- GMV and WMV were calculated from spatially normalized and modulated GM and WM maps (using CAT12) smoothed with 6 mm FWHM.
- Junction was derived from SPM12 segmentation results^1^.
- FLR was calculated from spatially a intensity normalized FLAIR scans^2^.
- Cortical thickness (Thick*) was extracted after T1 segmentation using CAT12 and smoothed with 15 FWHM surface-based Gaussian kernel^3^.
- Gyr*, SclD* ad Crtx* were extracted after T1 segmentation using CAT12 and smoothed with 25 FWHM surface-based Gaussian kernel.

**Statistical analysis**

Group statistics were calculated with a second-level model using SPM12. A two-sample t-test comparison of parameters files between patient and the healthy control group (N=110, sex and age matched) was performed; sex and age were included in GLM as nuisance variables. In case of modulated GM/WM maps multiplicative corrections with total intracranial volume were used.

**Transformation surface-based results map into MNI volume**

Surface based results (Thick*, Gyr*, SclD* and Crtx*) were transformed into a volume matrix for comparison purposes according Jianxiao^4^. (Only final results maps for comparison with the resection mask were transformed. The analysis itself, including comparisons with healthy controls, was performed in surface-based space.)

We are aware that the transformation of the surface base data into a voxel matrix brings limitations and therefore we marked these methods with an asterisk (*).

## MRI-IM: PCASL (ASLai, ASLhc)

**Data acquisition**

MRI data were acquired with a 3T scanner Siemens Prisma. Perfusion imaging was performed using pseudo-continuous ASL (PCASL) with single band EPI readout and following settings of MR scanner: Post labelling delay (PLD) 1800 ms, labelling duration (LD) 1800ms, slice readout time 30 ms, vx [3,3,6.6] mm, TE 16 ms, TR 4079 ms.

We acquired 21 pairs of tag and control images and M0 scan; M0 scan TR is 8000 ms.

**Protocol change**

At the beginning of the study, we used pulsed ASL (PASL), but during the study it found out that its results are insufficient (due to low SNR and technical shortcomings). Therefore, the sequence was changed to PCASL. 11 patients were measured with the new sequence, analyzed, and reported in this paper, we did not use older PASL data.

**Data processing**

Preprocessing of PCASL data was based on pipeline implemented in toolbox ExploreASL^5^ and consisted of motion correction, exclusion of outlying scans, registration to MNI space, qCBF quantification and partial volume correction. We excluded motion outliers using the *ENABLE*^6^ algorithm. qCBF quantification was calculated according^7^.

**Statistical analysis**

For ASL, we used two types of comparisons, both comparison with healthy controls (HC) and individual comparison of the right and left hemispheres using asymmetry index (AI)^8^. The image of ASL is strongly influenced by the cardiovascular conditions of an individual subject, which increases inter-subject variability. This effect can be compensated by comparing the right and left hemispheres, and due to the larger voxel size, the physiological differences between the hemispheres can be omitted.

- For ASLhc, we performed whole-brain voxel-wise two-sample t-test of qCBF perfusion maps comparing single patient to a group of 47 healthy controls (sex and age matched). Sex and age were used as covariates of no interest.
- ASLai was calculated based on voxel-wise asymmetry index (AI) of qCBF perfusion maps for identification of asymmetries in perfusion (left vs right hemisphere) according Lim et al. 2008 using formula: *ASLai = (qCBF_Lateral – qCBF_Contralateral) / (qCBF_Lateral + qCBF_Contralateral).* No other group statistics were used.

## MRI-IM: fMRI-rest (ALFFx, fALFFx, ReHoKCC)

**Data acquisition**

Calculation of Amplitude of low frequency fluctuations (ALFF) and fractional ALFF (fALFF) was based on resting-state BOLD fMRI data obtained during 20 minutes session of simultaneous EEG-fMRI recording as a part of whole epileptic protocol. The part of data (one third) was measured with TR of 2 s. Second third was measured with TR of 1 s and last third with TR of 600 ms. We evaluated possible effect of TR change on the results and there were no differences at p=0.05 uncorrected, thus we present the data together.

**Data processing**

Data were processed in Matlab and SPM12, accompanied with inhouse Matlab scripts. Preprocessing consisted of realign (motion correction), RETROICOR procedure to suppress physiological artifacts, coregistration of anatomical scans to BOLD data, spatial normalization using MNI template, and spatial smoothing using gaussian kernel with FWHM of 5 mm. Subsequently, very low frequencies were removed from BOLD data using high pass filter with cut off 128s, and other nuisance effects were regressed out of BOLD data - specifically white matter and CSF signals and six movement parameters obtained from realign procedure. Last step was masking BOLD data with grey matter mask obtained with SPM tissue probability map for grey matter with threshold of 0.2 (to remove outbrain voxels and left as much of GM voxels as possible).

The preprocessing quality was assessed by a visual inspection for low SNR and other artifacts, and *Framewise Displacement*^9^ was checked for fMRI motion artifacts.

**ALFF and fALFF calculation**

Such BOLD data was used for calculation of ALFF and fALFF. We used following frequency bands: (1) ALFF1/fALFF1: 0.01 - 0.027 Hz, (2) ALFF2/fALFF2: 0.027 - 0.073 Hz, (3) ALFF3/fALFF3: 0.073 - 0.198 Hz, (4) ALFF4/fALFF4: 0.198 - 0.3 Hz and (5) ALFF5/fALFF5: 0.3 - 0.5 Hz.

Fast Fourier transformation was applied on BOLD data to obtain power spectrum. ALFF was calculated as mean of power spectrum coefficients in specific band^10^. fALFF was calculated as sum of power spectrum coefficient in specific band divided by total BOLD signal energy (sum of all power spectrum coefficients)^11^.

**ReHo calculation**

Calculation of Regional Homogeneity (ReHo) was based on the same BOLD data as for ALFF/fALFF analysis. The only difference in preprocessing was omitting spatial smoothing to follow the standard ReHo processing pipeline as described in^12^. The ReHo coefficient in each voxel was calculated using KCC (Kendal concordance coefficient) as a metric of similarity. We used the set of 27 voxels, i.e. the BOLD signal time-courses from the center voxel and 26 surrounding voxels were used as input for the KCC calculation.

**Statistical analysis**

Group statistics were calculated with a second-level model using SPM12. A two-sample t-test comparison of parameters files between patient and the healthy control group (N=98, sex and age matched) was performed; sex and age were included in GLM as nuisance variables.

## MRI-IM: Diffusions (MD, AD, RD, FA, MK, AK, RK, MDK)

**Data acquisition**

Diffusion-weighted images (DWI) data acquisition was performed on Siemens 3T Magnetom Prisma using spin-echo echo-planar imaging sequence with following parameters: TR = 9000 ms, TE = 78 ms, FoV = 224 x 224 mm^2^, voxel size = 2.0 x 2.0 x 2.0 mm^3^ with a PAT factor of two. Diffusion weighting was performed in multi-directional diffusion weighting (MDDW) mode along 30 diffusion directions for each of 3 diffusion weightings: b_1_ = 700 s/mm^2^, b_2_ = 1000 s/mm^2^, b_3_ = 2300 s/mm^2^ and 10 directions for b_0_ = 0 s/mm^2^. Additionally, a ten non-diffusion weighted volume (b = 0 s/mm^2^) were acquired with opposite phase-encoding direction.

**Data processing**

Diffusion MRI data were preprocessed using MRtrix3.0^13^ and FMRIB Software Library (FSL) 6.0 (https://fsl.fmrib.ox.ac.uk/fsl/fslwiki). Preprocessing steps were set with the respect to recommendations reported in Maximov et al^14^., and consisted of following steps: (1) noise correction using Marchenko-Pastur principal component analysis (MP-PCA), (2) correction for Gibbs ringing artefact, (3) motion correction, eddy current and susceptibility distortion correction using FSL tools *eddy* and *topup*, (4) bias field correction calculated by Advanced Normalization Tools (ANTs)**.** (5) spatial smoothing using Gaussian kernel with the FWHM of 2.5 mm, as FWHM of 1.25 x voxel size (here 2mm) lead to higher anatomic specificity and (6) brain-only mask for each subject in a data analysis was estimated.

The preprocessing quality was assessed by visual inspection and automatically by using FSL’s tool *eddyqc*^15^. Z scores of SNR and CNR values were calculated, and their mean was used as a summary metric of data quality for each subject. The threshold of three standard deviations from the mean was set to assess low-quality preprocessing. The data of all subjects passed this cut-off and no data were excluded from our study because of SNR and/or CNR.

The preprocessed data were used to obtain parametric maps using DESIGNER toolbox^16^ involving following steps: (1) diffusion tensor was fitted for each voxel from DWI scans acquired with b_0_ and b_1_ values**,** and diffusional kurtosis tensor was fitted from scans measured with all b-values (i.e. b_1_ = 700 s/mm^2^, b_2_ = 1000 s/mm^2^, b_3_ = 2300 s/mm^2^, b = 0 s/mm^2^) for each voxel individually, (2) seven parameters were calculated in total mean diffusivity (MD), axial diffusivity (AD), radial diffusivity (RD), fractional anisotropy (FA) from diffusion tensor and mean kurtosis (MK), axial kurtosis (AK), and radial kurtosis (RK) from diffusional kurtosis tensor. Calculated maps were normalized into the Montreal Neuroscience Institute (MNI152).

**Statistical analysis**

The SPM12 software was used for statistical analysis. We performed whole-brain voxel-wise two-sample *t*-test of MD, AD, RD, FA, MK, AK and RK parametric maps comparing every single epileptic patient to a group of 100 healthy controls. Sex and age were used as covariates of no interest.

## PET-IM (PEThc, PETai)

**Data acquisition**

The PET images were acquired using a Siemens mCT Flow PET/CT scanner (4 detection rings with lutetium orthosilicate (LSO) type crystals and 22.1 cm axial field-of-view (FOV)). The intrinsic spatial resolution of the scanner was 4.5 mm at full width at half maximum (FWHM) 1cm from the centre of the FOV and 4.9 mm at full width at half maximum 10cm from the centre of the FOV. The dose of FDG administered was 170-200 MBq per subject with no weight differentiation. The emission acquisition time in 3-D mode was 10 minutes. Forty-seven tomographic slices with a 3-mm slice thickness were TrueX+TOF reconstructed with 400x400 iteration matrix with 5 iterations and 21 subsets and 2 mm FWHM Gaussian filter applied. Attenuation of PET images were corrected using LowDose CT with 3 mm slices.

Scans from each patient were visually checked for the correct physiological distribution of the radiotracer. Scans from patients who did not match the physiological distribution of the signal, e.g. extremely high signal values in the cerebellum, were ruled out from the analysis.

**Data processing**

The PET images were spatially normalized into the stereotactic MNI space using SPM8 (www.fil.ion.ucl.ac.uk) running under MATLAB 2011b and in-house template created as in Soma et al.^17^ and smoothed with gaussian spatial filter of 8 mm FWHM. The entire processing is described in detail in M. Kojan et al.^18^.

**Statistical analysis**

Similar as ASL, both comparison with healthy controls (HC) and individual asymmetry index (AI) were used.

- In PEThc case, we performed voxel-wise two-sample t-test of resulting metabolism maps comparing single patient to a group of 24 control subjects. Sex and age were used as covariates of no interest. The control subjects were recruited from oncological patients whose findings on their head FDG-PET were assessed as normal. They have normal MR scans and no neurological nor psychiatric diagnoses.
- PETai was calculated using formula for asymmetry index in the same way as ASLai (see MRI-IM: PCASL).

## SPECT-IM (SISCOM, ISAS, STATISCOM)

**Data acquisition**

The interictal and ictal SPECT images were acquired using two-head e-Cam Siemens machine equipped, with fan beam and LEHR collimators.

Scans from each patient were visually checked for the correct physiological distribution of the radiotracer. Scans from the patients who did not match the physiological distribution of the signal, e.g. radiotracer contamination on the skin, were ruled out from the analysis.

Fourteen ictal-interictal SPECT image pairs from healthy controls were downloaded from Healthy Normal Database on http://spect.yale.edu/downloads.html.

**Data processing**

We used Matlab and SPM12 together with simple MNI templates from SPM5 to process the data.

- SISCOM was calculated as follows: The two SPECT images (ictal and interictal) were coregistered and spatial normalized to the SPECT template available in SPM5. Images were subsequently smoothed with gaussian kernel with FWHM of 16 mm, masked using brainmask template available in SPM, and the mean intensity in each image was normalized to 100^19^.
- STATISCOM was calculated using the simple difference image (SISCOM), the mean difference from normal pairs of SPECT images and standard deviation from the same set of normal pairs. The SPECT images of healthy controls were processed in the same way as patient data for SISCOM. Subsequently mean and standard deviation was calculated for the differences between the pairs of normal SISCOM images^19^. The formula is:
   *STATISCOM = (diff_patient – mean(diff_normal)) / STD(diff_normal)*
- ISAS is the full statistical model set up in SPM using the two images from specific patient (ictal and interictal) and the set of pairs of SPECT images (two SPECT scans on subsequent days) from healthy controls. In each voxel, the difference between the ictal and interictal SPECT is evaluated by general linear model implemented in SPM and t-statistics is used for final evaluations^19^.

###

## EEG-IM: IEDs-dependent (ESI-HD)

**Data acquisition**

Data were recorded in shielded room with constant temperature. Subjects seated comfortably with eyes closed and were instructed to remain calm to avoid unnecessary movements. A HD EEG system with 256 channels (Electrical Geodesics, Inc.; GES 400 MR) was used with sampling frequency of 1kHz and Cz as the reference electrode. The impedance of each channel was always below 50kΩ. Twenty minutes of resting state data were acquired.

**Data processing**

The visual inspection of the data was performed to identify segments with artefacts and epileptiform discharges (IED). Two epileptologists trained in electrophysiology marked spikes during interictal period, which were consequently averaged after data preprocessing.

EEG recording was filtered to 1-40 Hz bandwidth with Butterworth filter of second order, 12dB/octave roll-off and forward and backward passes. Bad channels (0-4% of all channels) with artefacts were interpolated using spherical spline method from surrounding channels. Data were re-calculated to an average reference. Based on identified spike positions, data were segmented to <-500,500> ms intervals around spikes. Segments without artefacts were averaged.

Averaged spike activity was reconstructed into the source space using Cartool software complemented by an in-house solution running under MATLAB 2014b. T1-weighted subject specific MRI scan (measured without EEG cap) was used for forward Locally Spherical Model with Anatomical Constraints (LSMAC) model construction. Subject’s age was taken into consideration for optimal setup of volume conductivity. Low Resolution Electromagnetic Tomography (LORETA) was used for inversion modelling. Proper positions of EEG sensors were identified based on artefacts in another T1-weighted structural MRI scan, which was measured for this purpose with EEG cap before EEG recording in shielded room.

**Statistical analysis**

There was no further group statistical analysis, ESI data were evaluated individually.

## EEG-IM: IEDs-dependent (ESI-10-20)

**Data acquisition**

Data were recorded when subjects were monitored as inpatients in the Video EEG unit as a part of continuous video monitoring in St. Anne’s University Hospital at the First Department of Neurology.

An EEG system with 23 channels (10-20 system complemented by additional frontal and temporal channels) was used with sampling frequency of 1kHz and the reference electrode was created by average of Fz, Cz, Pz. The impedance of each channel was always below 50kΩ.

**Data processing**

The visual inspection of the data was performed to identify segments with artefacts and epileptiform discharges (IED). The epileptologist trained in electrophysiology marked spikes during interictal period, which were consequently averaged after data preprocessing.

EEG recording was filtered to 1-40 Hz bandwidth with Butterworth filter of second order, 12dB/octave roll-off and forward and backward passes. Data were re-calculated to an average reference. Based on identified spike positions, data were segmented to <-500,500> ms intervals around spikes. Segments without artefacts were averaged.

Averaged spike activity was reconstructed into the source space using Cartool software complemented by an in-house solution running under MATLAB 2014b. Montreal Neurological Institute (MNI) MRI template was used for forward Locally Spherical Model with Anatomical Constraints (LSMAC) model construction. Low Resolution Electromagnetic Tomography (LORETA) was used for inversion modelling.

**Statistical analysis**

There was no further group statistical analysis, ESI data were evaluated individually.

## EEG-IM: IEDs-independent (LocSyn)

**Data acquisition**

Data acquisition is same as in EEG-IM: IEDs-dependent (ESI-HD) section.

**Method**

The method is based on two studies that examined EEG data acquired by subdural electrodes in patients with pharmacoresistant partial epilepsy. The former study published by Schevon et al.^20^ showed increased synchrony between pairs of electrodes placed above seizure-onset-zone region when compared to other pairs. The effect persisted for a period of several days of repeated data acquisition. A former work of Warren et al.^21^ than published similar results showing that the synchrony is the most increased relative to bridging pairs of electrodes, i.e. electrodes inside and adjoining SOZ. The synchrony was even increased relative to normal tissue as shown with data from facial pain patients.

**Data processing**

We analysed HD EEG data. For each subject, we visually checked the data for common artifacts (e.g. muscles, eye-related artifacts, movements) and selected 300 one-second periods that contained no visible artifacts. The clean EEG data were projected into the source space using Cartool (ver. 3.6)^22^. We used individual T1 images, approx. 6000 solution points and all 256 electrodes. The exact positions of electrodes were estimated by semi-automatic analysis of fast T1 image acquired with EEG net on. Each solution-point EEG time course was projected into its average dipole orientation^23^. Then, we computed Corrected Imaginary Coherence for each pair of adjacent solution-points. The resulting map was transformed into the MNI space.

**Statistical analysis**

Finally, we computed a voxel-wise two-sample T-test using SPM12 toolbox to compare single patient’s data to a group of 87 healthy controls.

# Clinical sequences parameters

List of clinical sequences based on radiologists evaluated the (non)lesionality and determined the hypothesis. Measured on a Siemens 3T Prisma.

- **t2_tse_tra** – TR 6100 ms, TE 105 ms, voxel size 0.49x0.49x3 mm, flip angle 150 deg
- **t2_fl2d_tra_hemo** – TR 852 ms, TE 19.9 ms, vox 0.69x0x69x3.9 mm, flip angle 20 deg
- **t1_tir_tra** – TR 2500 ms, TE 11 ms, vox 0.69x0.69x3.3, flip angle 150 deg
- **t2_tirm_tra_dark-fluid** – TR 5390 ms, TE 81 ms, vox 0.69x0.69x3.6 mm, flip angle 150 deg
- **t1_tir_cor_p2** – TR 2900 ms, TE 10 ms, vox 0.5x0.5x2.2 mm, flip angle 150 deg
- **t2_tirm_cor_dark-fluid** – TR 9000 ms, TE 81 ms, vox 0.69x0.69x3.6 mm, flip angle 150 deg
- **t2_tse_cor_448_2mm_hippocampus** – TR 8000 ms, TE 52 ms, vox 0.39x0.39x2 mm, flip angle 150 deg
- **t1_mprage_sag_p2_iso** – TR 2300 ms, 2.33 ms, TI 900 ms, vox 1x1x1 mm, flip angle 8 deg
- **t1_mp2rage_sag_p2_iso** – TR 5000 ms, TE 2.97 ms, TI1 766 ms, TI2 2500 ms, vox 1x1x1 mm, flip angle1 4 deg, flip angle2 5 deg
- **t2_spc_FLAIR_sag_p2_iso** – TR 6000 ms, TE 387 ms, TI1 1900 ms, vox 1x1x1x mm

# Specificity

All in-brain voxels can be divided into four groups:

1. True Positive (TP) are voxels in the intersection of the surgery mask and the IM result.
2. False Negative (FN) are voxels inside the surgery mask, but outside the IM result.
3. False Positive (FP) are voxels outside the surgery mask, but inside the IM result.
4. True Negative (TN) are the remaining voxels outside the surgery mask and outside the IM result.

Other than that, the entire resection mask is Positive (P); *P = TP + FN*; and all voxels except the resection mask are Negative (N), where *N = FP + TN*. At the same time, it holds that P + N = 1 (whole in-brain volume).

After substituting into the specificity formula, we get

$$Specificity=SPC=\frac{TN}{N}=\frac{N-FP}{N}$$

We now substitute numerically. We know that the average mask size is 1% in-brain volume, which means that P = 0.01 and N = 0.99. The size of the IM result is always S_IM_ = 0.1 (because of the 90th percentile threshold). Then $SPC\cong\frac{0.99-FP}{0.99}$ and $FP\in\left[ S_{AIT}-P,S_{AIT} \right]=\left[ 0.09,0.1 \right]$. Finally, the resulting values of *SPC* are in interval $SPC\in\left[ 0.899,0.909 \right]\cong0.9\pm0.01$.

# Imaging methods (IMs) examples

*An overview of the results of all IMs in the resected area (marked by green contour) for patient 16 (ILAE 1, FCD IIB). The background is formed by a patient‘s T1 image normalized into MNI space. The individual IMs are described in Table 1.*

| **Method** | **Example** |
| --- | --- |
| GMV | 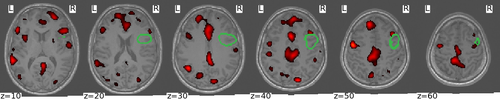 |
| GMC | 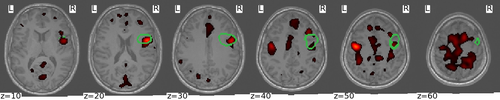 |
| Junction | 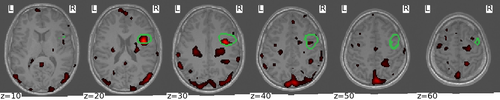 |
| Thick* | 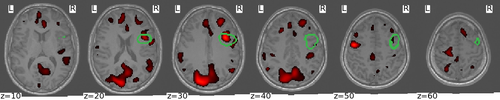 |
| SclD* | 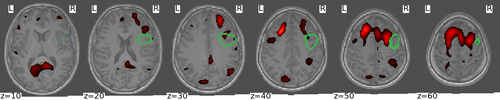 |
| Gyr* | 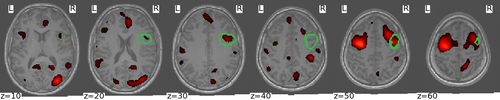 |
| Crtx* | 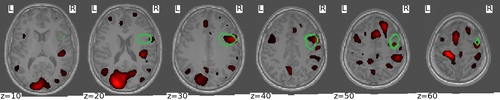 |
| WMV | 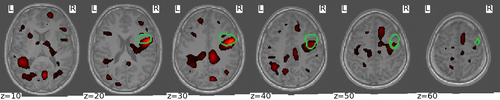 |
| WMC | 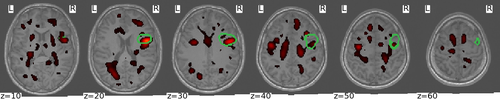 |
| FLR | 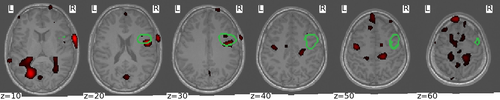 |
| ASLhc | 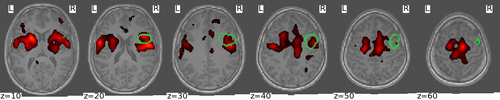 |
| ASLai | 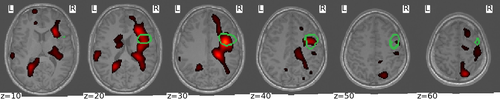 |
| ALFF1 | 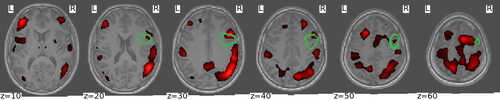 |
| ALFF2 | 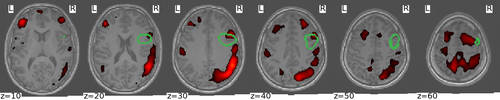 |
| ALFF3 | 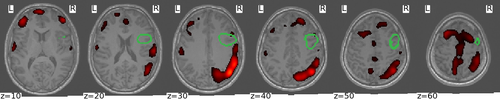 |
| ALFF4 | 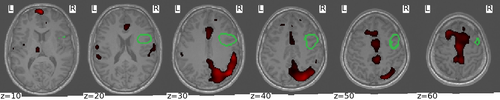 |
| ALFF5 | 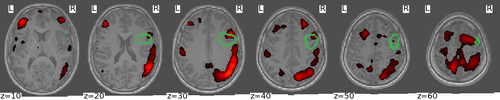 |
| fALFF1 | 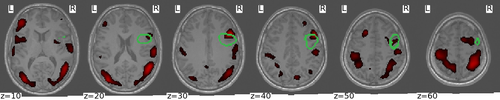 |
| fALFF2 | 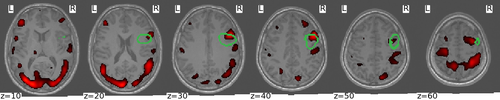 |
| fALFF3 | 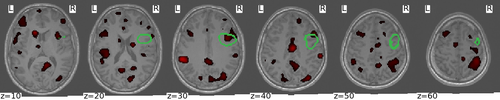 |
| fALFF4 | 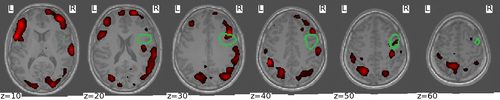 |
| fALFF5 | 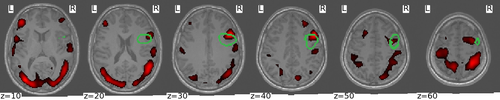 |
| ReHo | 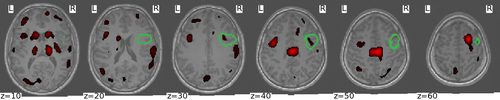 |
| MD | 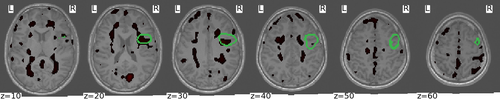 |
| AD | 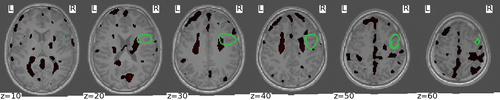 |
| RD | 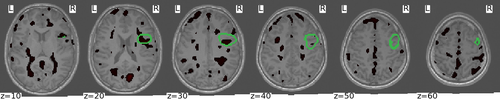 |
| FA | 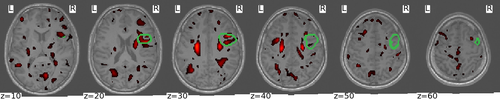 |
| MK | 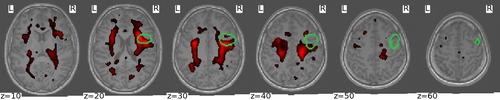 |
| AK | 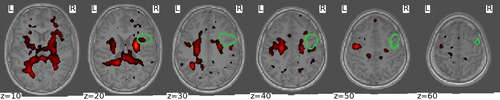 |
| RK | 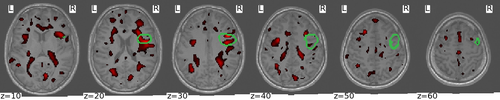 |
| PEThc | 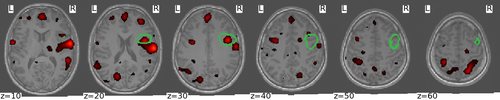 |
| PETai | 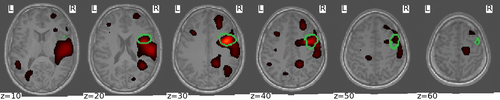 |
| SISCOM | 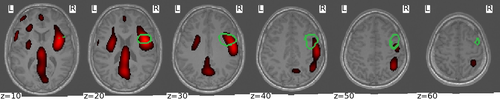 |
| STATISCOM | 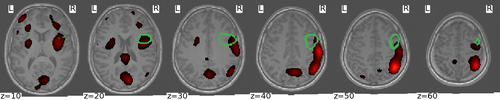 |
| ISAS | 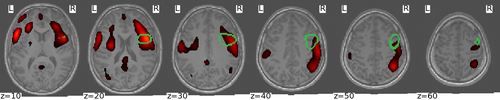 |
| ESI-HD | 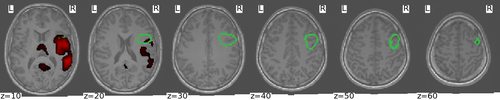 |
| ESI-10-20 | 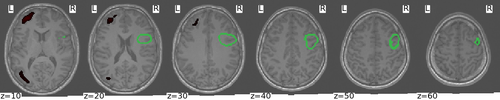 |
| LocSyn | 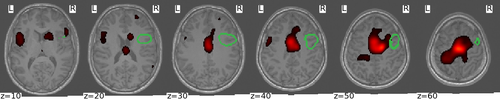 |

References

1. Huppertz, H. J. *et al.* Enhanced visualization of blurred gray-white matter junctions in focal cortical dysplasia by voxel-based 3D MRI analysis. *Epilepsy Res.* **67**, 35–50 (2005).

2. Focke, N. K. *et al.* Automated normalized FLAIR imaging in MRI-negative patients with refractory focal epilepsy. *Epilepsia* **50**, 1484–1490 (2009).

3. Dahnke, R., Yotter, R. A. & Gaser, C. Cortical thickness and central surface estimation. *Neuroimage* **65**, 336–348 (2013).

4. Jianxiao, W. U. *ACCURATE NONLINEAR MAPPING BETWEEN MNI152/COLIN27 VOLUMETRIC AND FREESURFER SURFACE COORDINATE SYSTEMS*. (2018).

5. Mutsaerts, H. J. M. M. *et al.* ExploreASL: An image processing pipeline for multi-center ASL perfusion MRI studies. *Neuroimage* **219**, 117031 (2020).

6. Shirzadi, Z. *et al.* Enhancement of automated blood flow estimates (ENABLE) from arterial spin-labeled MRI. *J. Magn. Reson. Imaging* **47**, 647–655 (2018).

7. Alsop, D. C. *et al.* Recommended implementation of arterial spin-labeled Perfusion mri for clinical applications: A consensus of the ISMRM Perfusion Study group and the European consortium for ASL in dementia. *Magn. Reson. Med.* **73**, 102–116 (2015).

8. Lim, Y. M. *et al.* Usefulness of pulsed arterial spin labeling MR imaging in mesial temporal lobe epilepsy. *Epilepsy Res.* **82**, 183–189 (2008).

9. Power, J. D., Barnes, K. A., Snyder, A. Z., Schlaggar, B. L. & Petersen, S. E. Spurious but systematic correlations in functional connectivity MRI networks arise from subject motion. *Neuroimage* **59**, 2142–2154 (2012).

10. Zang, Y. F. *et al.* Altered baseline brain activity in children with ADHD revealed by resting-state functional MRI. *Brain Dev.* **29**, 83–91 (2007).

11. Zou, Q. H. *et al.* An improved approach to detection of amplitude of low-frequency fluctuation (ALFF) for resting-state fMRI: Fractional ALFF. *J. Neurosci. Methods* **172**, 137–141 (2008).

12. Zang, Y., Jiang, T., Lu, Y., He, Y. & Tian, L. Regional homogeneity approach to fMRI data analysis. *Neuroimage* **22**, 394–400 (2004).

13. Tournier, J. D. *et al.* MRtrix3: A fast, flexible and open software framework for medical image processing and visualisation. *NeuroImage* vol. 202 116137 (2019).

14. Maximov, I. I., Alnæs, D. & Westlye, L. T. Towards an optimised processing pipeline for diffusion magnetic resonance imaging data: Effects of artefact corrections on diffusion metrics and their age associations in UK Biobank. *Hum. Brain Mapp.* **40**, 4146–4162 (2019).

15. Bastiani, M. *et al.* Automated quality control for within and between studies diffusion MRI data using a non-parametric framework for movement and distortion correction. *Neuroimage* **184**, 801–812 (2019).

16. Ades-Aron, B. *et al.* Evaluation of the accuracy and precision of the diffusion parameter EStImation with Gibbs and NoisE removal pipeline. *Neuroimage* **183**, 532–543 (2018).

17. Soma, T. *et al.* Usefulness of extent analysis for statistical parametric mapping with asymmetry index using inter-ictal FGD-PET in mesial temporal lobe epilepsy. *Ann. Nucl. Med.* **26**, 319–326 (2012).

18. Kojan, M. *et al.* Predictive value of preoperative statistical parametric mapping of regional glucose metabolism in mesial temporal lobe epilepsy with hippocampal sclerosis. *Epilepsy Behav.* **79**, 46–52 (2018).

19. Sulc, V. *et al.* Statistical SPECT processing in MRI-negative epilepsy surgery. *Neurology* **82**, 932–939 (2014).

20. Schevon, C. A. *et al.* Cortical abnormalities in epilepsy revealed by local EEG synchrony. *Neuroimage* **35**, 140–148 (2007).

21. Warren, C. P. *et al.* Synchrony in normal and focal epileptic brain: The seizure onset zone is functionally disconnected. *J. Neurophysiol.* **104**, 3530–3539 (2010).

22. Brunet, D., Murray, M. M. & Michel, C. M. Spatiotemporal analysis of multichannel EEG: CARTOOL. *Computational Intelligence and Neuroscience* vol. 2011 (2011).

23. Coito, A., Michel, C. M., Van Mierlo, P., Vulliemoz, S. & Plomp, G. Directed Functional Brain Connectivity Based on EEG Source Imaging: Methodology and Application to Temporal Lobe Epilepsy. *IEEE Trans. Biomed. Eng.* **63**, 2619–2628 (2016).
